# Long-Term Land Use Affects Phosphorus Speciation and the Composition of Phosphorus Cycling Genes in Agricultural Soils

**DOI:** 10.3389/fmicb.2018.01643

**Published:** 2018-07-20

**Authors:** Jin Liu, Barbara J. Cade-Menun, Jianjun Yang, Yongfeng Hu, Corey W. Liu, Julien Tremblay, Kerry LaForge, Michael Schellenberg, Chantal Hamel, Luke D. Bainard

**Affiliations:** ^1^College of Agronomy and Biotechnology, China Agricultural University, Beijing, China; ^2^Visiting Scientist, Agriculture and Agri-Food Canada, Swift Current Research and Development Centre, Swift Current, SK, Canada; ^3^Swift Current Research and Development Centre, Agriculture and Agri-Food Canada, Swift Current, SK, Canada; ^4^Institute of Environment and Sustainable Development in Agriculture, Chinese Academy of Agricultural Sciences, Beijing, China; ^5^Canadian Light Source, University of Saskatchewan, Saskatoon, SK, Canada; ^6^Stanford Magnetic Resonance Laboratory, Stanford University School of Medicine and ChEM-H-Stanford University, Stanford, CA, United States; ^7^Energy, Mining and Environment, National Research Council of Canada, Montreal, QC, Canada

**Keywords:** land use, soil, phosphorus, solution NMR, XANES, shotgun metagenomics

## Abstract

Agriculturally-driven land transformation is increasing globally. Improving phosphorus (P) use efficiency to sustain optimum productivity in diverse ecosystems, based on knowledge of soil P dynamics, is also globally important in light of potential shortages of rock phosphate to manufacture P fertilizer. We investigated P chemical speciation and P cycling with solution ^31^P nuclear magnetic resonance, P K-edge X-ray absorption near-edge structure spectroscopy, phosphatase activity assays, and shotgun metagenomics in soil samples from long-term agricultural fields containing four different land-use types (native and tame grasslands, annual croplands, and roadside ditches). Across these land use types, native and tame grasslands showed high accumulation of organic P, principally orthophosphate monoesters, and high acid phosphomonoesterase activity but the lowest abundance of P cycling genes. The proportion of inositol hexaphosphates (IHP), especially the *neo*-IHP stereoisomer that likely originates from microbes rather than plants, was significantly increased in native grasslands than croplands. Annual croplands had the largest variances of soil P composition, and the highest potential capacity for P cycling processes based on the abundance of genes coding for P cycling processes. In contrast, roadside soils had the highest soil Olsen-P concentrations, lowest organic P, and highest tricalcium phosphate concentrations, which were likely facilitated by the neutral pH and high exchangeable Ca of these soils. Redundancy analysis demonstrated that IHP by NMR, potential phosphatase activity, Olsen-P, and pH were important P chemistry predictors of the P cycling bacterial community and functional gene composition. Combining chemical and metagenomics results provides important insights into soil P processes and dynamics in different land-use ecosystems.

## Introduction

Driven by the increasing demand for agricultural production, land-use change has been widespread globally over the last several decades (Guillaume et al., 2015). In most parts of the world, the original vegetation has been cleared for the expansion of croplands and pastures, both of which are typical land uses crucial for food production (Houghton, 1994). Agricultural areas mostly devoted to either arable croplands or grazed pastures comprise about one third of the land surface globally (FAO/UNEP, 2015). The global effects of land-use change also contribute to global changes in nutrient cycling and dynamics (Houghton, 1994). This is especially significant for phosphorus (P), which often limits the productivity and sustainability of agriculture, requiring fertilization. Rock phosphate sources used to produce fertilizers are globally limited, and there are concerns about their long-term availability (Elser and Bennett, 2011; Sharpley et al., 2013). Additionally, P loss from agriculture can have a negative effect on the aquatic environment. This is expected to continue even if P fertilization is reduced due to the large amount of residual P accumulated in agricultural soils through time in many regions (Garcia-Montiel et al., 2000; Sharpley et al., 2013; Stutter et al., 2015). Efficient P use is therefore a priority when replacing natural ecosystems with managed ecosystems (Stutter et al., 2015).

Conversion of land-use is expected to change soil P dynamics. Changes in P inputs and outputs through management practices that alter soil physical, chemical and biological properties affect the chemical nature of different P species in soils and ultimately their bioavailability (Condron et al., 2005; Maranguit et al., 2017). A full understanding of the effects of land-use and management systems on soil P composition remains obscure, partially due to methodological limitations such as a reliance on the widely used but operationally defined sequential fractionation approach (Guggenberger et al., 1996; Negassa and Leinweber, 2009; Crews and Brookes, 2014; Maranguit et al., 2017). A more useful method, capable of identifying P species, particularly organic P (P_o_) compounds, in soils at the molecular level, is solution ^31^P nuclear magnetic resonance (P-NMR) spectroscopy. With P-NMR, more detailed insights into P_o_ species behind the P pools have been revealed in recent years (Condron et al., 2005; McDowell and Stewart, 2006; Stutter et al., 2015). Nevertheless, many published studies have limited peak identification to clearly separated, distinct peaks only (e.g., orthophosphate and pyrophosphate) and have grouped the remaining peaks together into broad compound classes such as orthophosphate monoesters and diesters; this provides limited information about P cycling and availability (Cade-Menun, 2017). Additionally, even within a single broad category such as orthophosphate monoesters or orthophosphate diesters, P_o_ forms differ in their bioavailability and reactivity. As such, a full understanding of P cycling in soils requires identifying as many specific P_o_ species as possible (Cade-Menun, 2015; Cade-Menun, 2017). Additionally, P K-edge X-ray absorption near-edge structure (P-XANES) spectroscopy provides a new and powerful approach to directly identify inorganic P (P_i_) compounds (Prietzel et al., 2013; Liu et al., 2015). Therefore, the combined application of P K-edge XANES and P-NMR spectroscopy allows for a comprehensive identification of soil P species across ecosystems with different land uses, to an extent not accomplished with previous studies.

Land use change will introduce significant changes in vegetation, which can alter soil biology and nutrient cycling. Vegetation changes will change rooting depth, nutrient and water uptake, soil chemistry and symbioses, such as N fixation or mycorrhizae (Bainard et al., 2017; Cade-Menun et al., 2017a). Plants and microorganisms are essential drivers of soil P turnover and dynamics. They enhance the solubilization of P by the release of low molecular weight acids and phosphatases that mineralize P_o_ (Richardson et al., 2011). Under conditions of phosphate deficiency, bacteria can induce the phosphate (Pho) regulon to excrete phosphatases to obtain bioavailable orthophosphate (Santos-Beneit, 2015). Land use has been shown to influence specific functional genes (e.g., phytase and phosphatase genes), and the composition of microbial communities associated with P cycling in soils (Jangid et al., 2008; Neal et al., 2017). Few studies to date have generated a thorough insight into the response of soil microbial communities and their P cycling capacity, coupled with soil P chemistry (e.g., soil physico-chemical parameters and P speciation) to land use.

The general objective of this study was to investigate the effects of land use change on P cycling, using advanced chemical techniques and metagenomics sequencing. We chose four agricultural areas in southwestern Saskatchewan, with adjacent sites of four typical agricultural land uses in the region: annual cropland, native grassland, tame grassland and roadsides. The close proximity of these locations and their historical continuity of land use (each > 50 years) kept all otherwise interrelated variables relatively constant (climate, topography, parent material, etc.) except land use. Samples from the four locations were patterned as replicated sites for each land use, providing a unique and valuable research platform to clarify soil P cycling induced by land use change. Combining state-of-the-art spectroscopic approaches (solution P-NMR and P K-edge XANES spectroscopy) with metagenomics, the specific objectives of this study were: (1) to characterize the composition of P_i_ and P_o_ in the soils under various land uses; (2) to investigate the abundance and composition of P functional genes and the microbial community within various ecosystems; and (3) to link these chemical and metagenomics results together for a better understanding of P cycling processes under different land uses.

## Materials and Methods

### Field Sites and Sample Collection

The four experimental sites [Auvergne Wise Creek (AWC), Val Marie (VM), Masefield (MF1, MF2)], located in southwestern Saskatchewan, have the same soil type (well-drained Orthic Brown Chernozems; Saskatchewan Environment, and Resource Management [SERM], 1997) and a known history of more than 50 years in each studied land use type (Cade-Menun et al., 2017a). The native grasslands were a mixed grass prairie community, and tame grasslands were crested wheatgrass [*Agropyron cristatum* (L.) Gaetern.] stands that had been established for over 50 years. The native and tame grasslands used for this study were in pastures grazed for beef production. Croplands were in dryland (unirrigated) annual wheat-based production. The fourth land use type in this study was roadside ditches, which serve as buffers between roads and fields. More details of the experimental sites and the broader land use study are available in Cade-Menun et al. (2017a). For the current study, soil samples were collected in July 2013 from the 0 to 30 cm depth from four land use types at four locations (*n* = 16). At each location, six soil cores (1.9 cm diameter) were collected inside four 1 m^2^ quadrats that were situated along a 10 m transect for a total of 24 total soil cores per location. This differs from Cade-Menun et al. (2017a) with respect to date, sampling depth and number of study sites. Soil samples were stored in a cooler with ice packs while in the field. At the lab, field-moist soil cores from each location were pooled together and sieved (<2 mm) to form one composite sample per location and to remove rocks, larger roots, and coarse plant material. A portion of each composite soil sample was air-dried and stored at room temperature for chemical analysis, including XANES. An additional sub-sample was immediately stored at -20°C for molecular analysis, and the remainder was refrigerated (4°C) for enzyme assays and extraction for P-NMR.

### Chemical Analysis

Soil pH was measured in CaCl_2_ (1:2 w/v; Hendershot et al., 2008). Soils were analyzed for total C, total N, and organic C (after acidification) by dry combustion (Vario Micro Cube, Elementar). Total P was determined by digestion (Parkinson and Allen, 1975), total P_o_ was determined by the ignition method (Saunders and Williams, 1955), and Olsen-P was determined with sodium bicarbonate extraction (Sims, 2009), all followed by colorimetric analysis (Murphy and Riley, 1962). Mehlich-3 extraction (Sims, 2009) was used to determine P, Al, Ca, and Fe through analysis of extracts by inductively coupled plasma optical emission spectroscopy (ICP-OES; Thermo Scientific ICAP 6300 Duo). Exchangeable Ca was extracted in ammonium acetate and measured by ICP-OES (Hendershot et al., 2008). The activities of acid and alkaline phosphomonoesterase were assayed with *p*-nitrophenyl phosphate as substrates with the buffer pH-values adjusted to 6.5 and 11, respectively, phosphodiesterase activity was assayed with *bis-p*-nitrophenyl phosphate as substrate with the buffer pH 8.0 (Tabatabai, 1994).

### Solution P-NMR Spectroscopy

Refrigerated soils were extracted with NaOH-EDTA in a 1:10 soil: extract ratio for P-NMR as previously described (Liu et al., 2015). Solution P-NMR spectra were collected as described in Cade-Menun et al. (2017b), using a Varian INOVA 600 MHz (202.5 MHz for P) spectrometer with a 10 mm broadband probe at the Stanford Magnetic Resonance Laboratory. The NMR parameters were: 90° pulse (30 μs), 0.675 s acquisition time, 4.32 s pulse delay, 20°C, 2,160–11,520 scans (3–16 h); no proton decoupling. The delay time used was based on the P:(Fe + Mn) concentrations in extracts (McDowell et al., 2006; Cade-Menun and Liu, 2014). To facilitate peak identification, spiking experiments with phytate, α- and β-glycerophosphate and adenosine monophosphate were conducted (Cade-Menun, 2015; Liu et al., 2015; Cade-Menun et al., 2017b). Compounds were identified by their chemical shifts after the orthophosphate peak in each spectrum was standardized to 6.0 ppm during processing. Peak areas were calculated by integration on spectra processed with 7 and 2 Hz line-broadening, using NUTS software (2000 edition; Acorn NMR, Livermore, CA, United States) and manual calculation. Percentages of orthophosphate monoesters and diesters were corrected for degradation of diesters to monoesters during NMR analysis (Liu et al., 2015; Cade-Menun et al., 2017b).

### Phosphorus K-Edge XANES Spectroscopy

Phosphorus K-edge XANES spectra were collected at the Soft X-ray Micro-characterization Beamline (SXRMB) equipped with a InSb(111) double-crystal monochromator at the Canadian Light Source (CLS), Saskatoon, SK, Canada. Detailed information on instrument setting, sample preparation and data collection was described previously (Liu et al., 2014, 2015). In brief, soil samples were thinly spread over a P-free and double-sided carbon tape for the XANES measurements. The soil spectra were collected in partial fluorescence yield (PFY) mode using a four-element fluorescence detector. At least three XANES spectra were collected and averaged for each soil sample to obtain acceptable signal-to-noise level. Radiation damage during XANES experiment was excluded by a good reproducibility of the repeated measurements on the same spot and repeated scans over different spots for each sample. All XANES spectra were analyzed by Athena (Ravel and Newville, 2005). The absolute energy scale was calibrated to 2,149 eV (E_0_) as the maximum energy of the first peak in the first derivative spectrum of AlPO_4_ (Beauchemin et al., 2003). Spectra were background corrected by a linear regression fit through the pre-edge region and normalized total K-edge intensity to one unit edge jump by defining the continuum regions (>50 eV above absorption edge) as the post-edge region. Principal component analysis (PCA) was performed on the set of 16 soil XANES spectra using the program SixPack (Webb, 2005). According to PCA results (Supplementary Table S1), the minimum indicator (IND) suggested that four components were optimal for linear combination fitting (LCF) analysis of these soil samples. Consistently, the variations of the fifth component almost represented random variations due to noise rather than real spectral variations (Supplementary Figure S1). As there were up to four components contributing to 92.5% of spectra variations for all of the investigated samples (Supplementary Table S1 and Supplementary Figure S1), LCF of soil spectra were performed over the spectral energy region from 2,139 to 2,164 eV using all possible binary, ternary, and quaternary combinations of our reference spectra which were collected at the same beamline and reported in our previous studies (Liu et al., 2013, 2015). The E_0_ was fixed during LCF analysis and weights of all P standards used were forced to sum 1. Phosphorus forms with proportions <10% were excluded from the fit set and replaced with other possible P species (Weyers et al., 2016). The goodness-of-fit was judged by the Chi-squared values and *R* values, and P standards yielding the best fit were considered as the most possible P species in the investigated soil samples (Supplementary Figure S2 and Supplementary Table S2).

### Metagenomic Analysis

Total nucleic acids were extracted from 1 g (2 × 0.5 g) frozen soil from each sample using the PowerSoil DNA Isolation Kit (Mo Bio Laboratories, Carlsbad, CA, United States) following the manufacturer’s recommended protocol. The DNA samples were quantified using the Qubit dsDNA BR Assay Kit (ThermoFisher Scientific) and 2,100 Bioanalyzer instrument. Metagenomic libraries were prepared and sequenced on an Illumina HiSeq2500 system on a rapid mode 2 × 150 bp configuration. A total of 20 samples were submitted for metagenome sequencing of which the resulting data (273 Giga-bases) were processed through our metagenomics bioinformatics pipeline (Tremblay et al., 2017). Read count summaries and mapping statistics are provided (Supplementary Table S3). Sequencing adapters were removed from each read and bases at the end of reads having a quality score <30 were cut off (Trimmomatic v0.32; Bolger et al., 2014) and scanned for sequencing adapters contaminants reads using DUK^1^ to generate quality controlled (QC) reads. The QC-passed reads from each sample were co-assembled using Megahit v1.1.2 (Li et al., 2015) on a 3 Tera-Bytes of RAM compute node with iterative kmer sizes of 31, 41, 51, 61, 71, 81, and 91 bases (see Supplementary Table S4 for assembly statistics). Gene prediction was performed by calling genes on each assembled contig using Prodigal v2.6.2 (Hyatt et al., 2010). Genes were annotated following the JGI’s guidelines (Huntemann et al., 2015) including the assignment of KEGG orthologs (KO; Kanehisa and Goto, 2000). The QC-passed reads were mapped (BWA mem v0.7.15^2^) against contigs to assess quality of metagenome assembly and to obtain contig abundance profiles. Alignment files in bam format were sorted by read coordinates using samtools v1.2 (Li et al., 2009), and only properly aligned read pairs were kept for downstream steps. Each bam file (containing properly aligned paired-reads only) was analyzed for coverage of called genes and contigs using bedtools (v2.17.0; Quinlan and Hall, 2010) using a custom bed file representing gene coordinates on each contig. Only paired reads both overlapping their contig or gene were considered for gene counts. Coverage profiles of each sample were merged to generate an abundance matrix (rows = contig, columns = samples) for which a corresponding CPM (Counts Per Million–normalized using the TMM method; edgeR v3.10.2; Robinson et al., 2010). Taxonomic summaries were performed using a combination of in-house Perl and R scripts and Qiime v.1.9.1 (Caporaso et al., 2010).

### Statistical Analyses

One-way ANOVAs were conducted for all the data of each land use (*n* = 4) separately, using SPSS 13.0 (SPSS, Inc), followed by a least significant difference (LSD) test with α = 0.05. The P-NMR data were clr transformed prior to statistical analysis (Abdi et al., 2015; Liu et al., 2015); other data were transformed as needed for normality. Redundancy analysis (RDA) was used to identify the important P chemistry-related drivers of the P-cycling soil bacterial community and functional gene composition. A set of nonredundant predictors of the bacterial community and functional gene composition were selected using the *ordistep* and *envfit* functions (*vegan* package, R 3.4.3). Only the significant variables were included in the final models, excluding collinear variables with a variance inflation factor >10.

## Results

### Soil Properties

Among the investigated land uses, there were no significant differences in soil pH, total P, total C, organic C, total N, Mehlich-extractable P, Al, Fe, and Ca (**Table 1**). The percentage of P_o_ was significantly higher for both native and tame grasslands (both 75.4%) than for annual croplands (52.6%) and roadside soils (54.4%, **Table 1**). The highest Olsen-P concentration was observed in roadside soils (12.5 mg kg^-1^), which was significantly higher than native grasslands (3.8 mg kg^-1^) and annual croplands (5.6 mg kg^-1^, **Table 1**). The highest exchangeable Ca occurred in roadside soils and the lowest in tame grasslands (**Table 1**).

**Table 1:** Selected physiochemical properties of the soils under different land uses (means ± standard errors, *n* = 4)^a^.

Land uses	pH	Total P	Organic P	Total C	Org C	Total N	Olsen-P	Mehlich P	Mehlich Al	Mehlich Fe	Mehlich Ca	NH_4_OAc extracted Ca
	
		mg kg^-1^	%	%	%	%				mg kg^-1^		
Roadside soils	7.1 ± 0.1 a	479.3 ± 46.4 a	54.4 ± 2.6 b	2.2 ± 0.2 a	1.6 ± 0.2 a	0.15 ± 0.03 a	12.5 ± 3.9 a	22.8 ± 6.4 a	373.8 ± 106.5 a	140.6 ± 37.1 a	5223.3 ± 188.9 a	3698.4 ± 100.7 a
Native grasslands	6.6 ± 0.2 a	381.3 ± 24.3 a	75.4 ± 3.2 a	2.1 ± 0.3 a	1.7 ± 0.2 a	0.18 ± 0.03 a	3.8 ± 0.4 b	15.0 ± 2.9 a	532.0 ± 63.0 a	97.1 ± 7.0 a	3250.4 ± 1130.1 a	2446.1 ± 496.5 ab
Tame grasslands	6.3 ± 0.3 a	401.2 ± 42.7 a	75.4 ± 8.0 a	1.9 ± 0.2 a	1.7 ± 0.2 a	0.18 ± 0.03 a	6.9 ± 1.8 ab	19.0 ± 4.2 a	630.0 ± 138.5 a	127.6 ± 24.4 a	2662.5 ± 772.2 a	1759.4 ± 290.8 b
Annual croplands	7.1 ± 0.3 a	381.1 ± 32.9 a	52.6 ± 8.3 b	2.0 ± 0.3 a	1.2 ± 0.4 a	0.14 ± 0.07 a	5.6 ± 2.6 b	12.1 ± 5.3 a	372.2 ± 163.3 a	81.0 ± 18.8 a	6037.0 ± 2403.1 a	3006.9 ± 732.6 ab

*^a^Values in each column followed by the same lowercase letters are not significantly different according to LSD (P < 0.05).*

### Solution P-NMR Spectroscopy and Soil Phosphatase Activities

**FIGURE 1:**
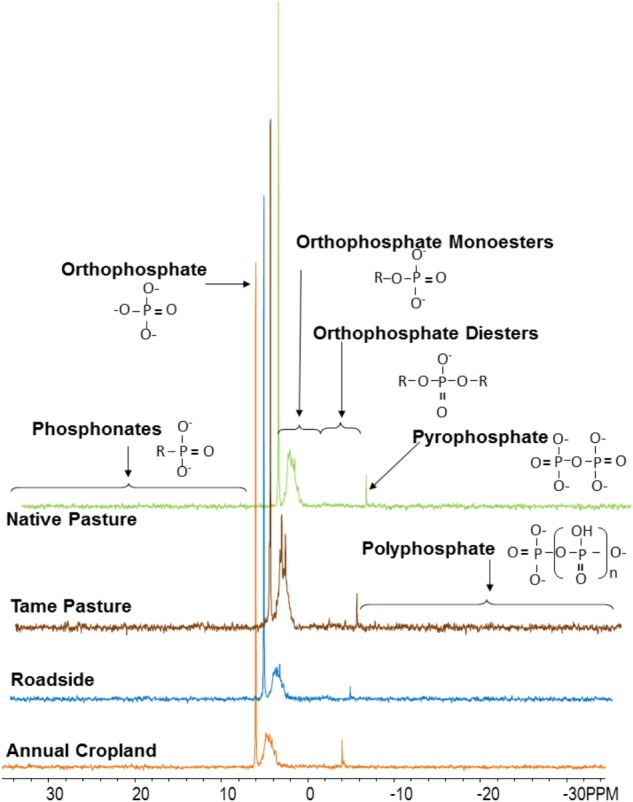
Spectra (P-NMR) for each land use type from the Masefield 1 location. Spectra were processed with 7 Hz line-broadening, and are plotted to full height and scaled to the height of the orthophosphate peak.

Examples of P-NMR spectra for these samples are shown in **Figure 1** and Supplementary Figure S3; chemical shifts of identified peaks are shown in Supplementary Table S5, and proportions of P forms and compound classes determined by P-NMR are shown in **Table 2** and Supplementary Table S6. Extraction with NaOH-EDTA recovered 34.9–50.5% of total P without significant differences among the land use types (**Table 2**). Inorganic P in the NaOH-EDTA extracts was significantly greater in roadside samples (46.3%) than native and tame grasslands (31.5 and 30.9%; **Table 2**), and was mainly orthophosphate (27.8–43.1%) with traces of polyphosphate (2.2–2.4%) and pyrophosphate (0.7–1.3%, Supplementary Table S6). The percentage of phosphonates was low (1.3–1.8%) with no differences among land use types. There were no significant differences among land use types for orthophosphate diesters with (CDiest, 19.5–24.0%) and without (Di, 2.5–4.1%) correction for degradation products (Deg, **Table 2**). In these samples, diester degradation was primarily from RNA to mononucleotides (Nucl, Supplementary Table S6), because the percentages of α- and β-glycerophosphates (degradation products of phospholipids) were low, and peaks were identified in the OthDi1 category where undegraded phospholipids would be observed (Supplementary Table S6). There were significant differences among the land use types for orthophosphate monoesters, with (Mono) and without (Cmono) correction for degradation (**Table 2**), with native and tame grasslands greater than roadside and annual cropland samples.

**Table 2:** Phosphorus form classes or ratios of form classes^a^ determined by ^31^P nuclear magnetic resonance spectroscopy for the studied soil samples under different land uses (means ± standard errors, *n* = 4)^b^.

Land uses	Recovery	P_i_	P_o_	TotPoly	IHP	Myo:other	Mono	Di	M:D	Cmono	CDiest	Deg	CM:D
Roadside	34.9 ± 3.6 a	46.3 ± 5.7 a	53.7 ± 5.7 b	3.2 ± 0.7 a	13.8 ± 1.0 b	0.4 ± 0 ab	48.6 ± 4.5 b	3.7 ± 1.8 a	20.9 ± 6.5 a	32.8 ± 3.3 c	19.5 ± 2.5 a	15.8 ± 1.7 a	1.7 ± 0.1 a
Native grasslands	43.1 ± 2.4 a	31.5 ± 2.6 b	68.5 ± 2.6 a	3.7 ± 0.4 a	18.4 ± 1.2 ab	0.3 ± 0.1 ab	63.1 ± 2.4 a	3.9 ± 0.9 a	19.3 ± 4.9 a	43.0 ± 1.2 ab	24.0 ± 1.8 a	20.1 ± 1.4 a	1.8 ± 0.1 a
Tame grasslands	50.5 ± 6.6 a	30.9 ± 2.7 b	69.2 ± 2.7 a	3.1 ± 0.2 a	24.9 ± 3.8 a	0.2 ± 0 b	64.9 ± 2.8 a	2.5 ± 0.6 a	31.6 ± 7.8 a	46.7 ± 3.8 a	20.7 ± 1.9 a	18.2 ± 2.4 a	2.4 ± 0.4 a
Annual crop	47.6 ± 6.7 a	41.4 ± 2.3 ab	58.6 ± 2.3 ab	3.0 ± 0.6 a	16.8 ± 2.3 b	0.4 ± 0.1 a	53.2 ± 1.7 b	4.1 ± 0.7 a	13.7 ± 1.6 a	35.9 ± 1.9 bc	21.4 ± 0.8 a	17.3 ± 0.7 a	1.7 ± 0.1 a

*^a,b^Values in each column followed by the same lowercase letters are not significantly different according to LSD (P < 0.05). P_i_, inorganic P; P_o_, organic P; TotPoly, total polyphosphate, IHP, inositol hexakisphosphate; Myo:other, the ratio of myo-IHP to other IHP species; Mono, orthophosphate monoester; Di, orthophosphate diesters; M:D, the ratio of orthophosphate monoester to orthophosphate diesters; Deg, degradation; C denotes a correction for degradation products.*

By NMR, P_o_ represented 53.7–69.2% of the NaOH-EDTA extracted P in the tested soils (**Table 2**). The greatest P_o_ proportions were in native and tame grasslands (**Table 2**) and the least was in roadside soils, consistent with general soil chemical results (**Table 1**). The significant differences among the land use types for P_o_ predominantly occurred through differences in inositol hexakisphosphates (IHP, **Table 2**) and an unidentified P compound resolved at 4.9 ppm (Supplementary Tables S5, S6). Among the four stereoisomers of IHP, the 4 equatorial/2 axial configuration of *neo*-IHP in native grasslands (1.1%) was significantly higher than that in annual croplands (0.6%). The unidentified peak at 4.9 ppm, which was significantly higher for tame and native grasslands, may be the 4 axial/2 equatorial configuration of *neo*-IHP (Turner et al., 2012), but this could not be confirmed with spiking. There were no significant differences among other land use types for the other IHP stereoisomers (Supplementary Table S6), or for the other P_o_ compound classes and identified species, although some of them showed higher proportions in grasslands than other land uses (**Table 2** and Supplementary Table S6).

Phosphatases play a key role in catalyzing the hydrolysis of P_o_ to release orthophosphate for plant uptake. The activity of acid phosphomonoesterase, produced by plants and microbes (Tabatabai, 1994), was significantly lower in soils from annual cropland than other land use types (Supplementary Figure S4). There were no significant differences among land use types for alkaline phosphatase, which is produced by microbes only, and the activity of this enzyme was lower than that of acid phosphatase for all but annual cropland soils. Phosphodiesterase activities were lowest for land use types compared to phosphomonoesterase activities, and were significantly lower in annual cropland soils than roadside soils.

### Phosphorus K-Edge XANES Spectroscopy

**FIGURE 2:**
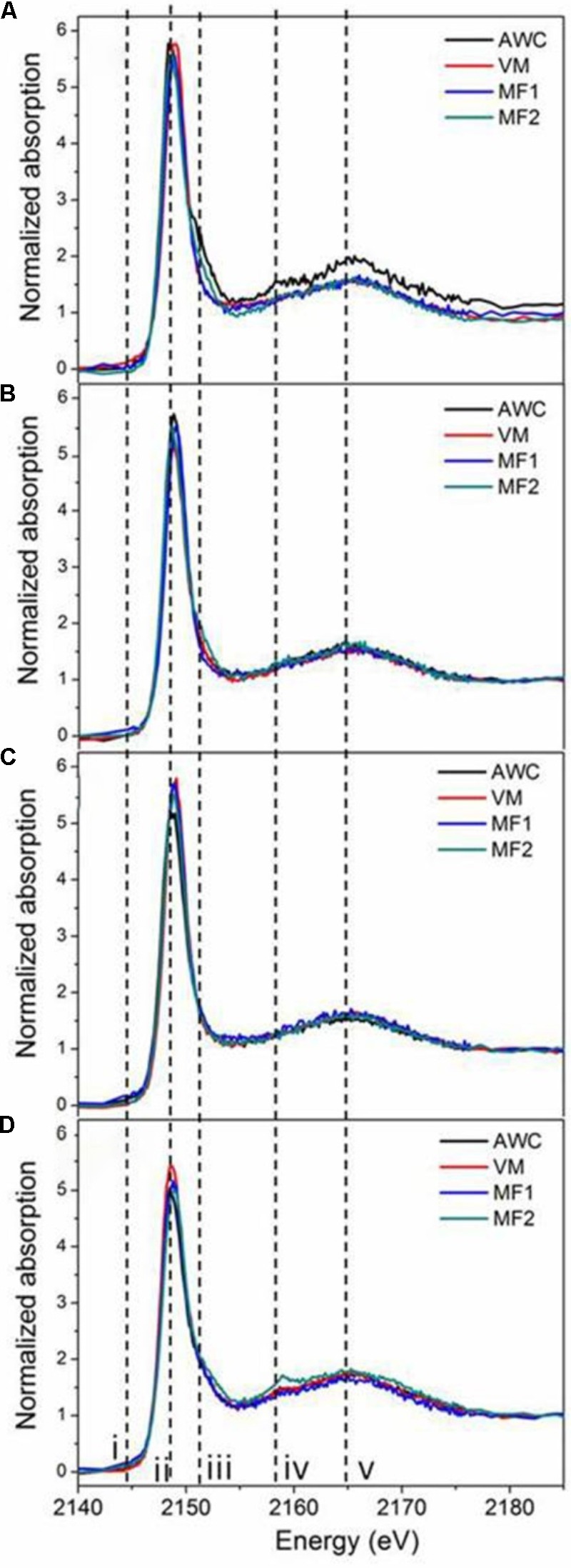
P K-edge XANES spectra of soils under different land uses. Figure panels are for soils from annual croplands **(A)**, tame grasslands **(B)**, native grasslands **(C)**, roadsides **(D)**. Samples from each land use type were collected at four locations: Val Marie (VM), Auvergne Wise Creek (AWC), Masefield1 (MF1), and Masefield2 (MF2).

Supplementary Figure S5 shows the K-edge XANES spectra of P standards in this study, where similar shapes with both the white line peak (ii) and oxygen oscillation peak (v) are observed. In addition, these P standards exhibited fingerprinting features that allow for identification and quantification of different P species in soil samples. For example, all Ca-associated P (Ca-P) standards exhibit a post-edge shoulder (iii) that is sharper for compounds containing many Ca atoms [HAP and Ca_3_(PO_4_)_2_] than for monocalcium phosphates [Ca(H_2_PO_4_)_2_ and CaHPO_4_], and another signal (iv). Iron phosphate (FePO_4_) displayed a pre-edge feature (i). Aluminum phosphate (AlPO_4_), without the pre-edge feature (i) seen for FePO_4_, shows a post-edge feature (iv) similar to Ca-P. These features agree well with previous reports (Beauchemin et al., 2003; Lombi et al., 2006; Prietzel et al., 2013). Spectra of native and grassland soils (**Figures 2C,B**), rather similar across locations, resembled the spectra of IHP (Supplementary Figure S5). As the K-edge XANES spectra of P_o_ compounds are mostly featureless and hard to differentiate, the contribution of IHP is interpreted as the presence of all possible organically bound P rather than specifically to IHP in the samples. Roadside soils showed spectra with a broad post-edge feature and slight shoulders (iii and iv, **Figure 2D**), demonstrating the presence of Ca-P in these soils. In contrast, spectra of cropland soils showed more variation than those of other land uses (**Figure 2A**).

The LCF for each soil sample, shown in Supplementary Figure S2 and Supplementary Table S2, indicated that IHP was present in all soil samples, in greater proportions for the native (92%) and tame (82%) grasslands than that for the roadside soils (50%, **Table 3**). The roadside soils from all four locations also contained some Ca-P in the form of tricalcium phosphate (TCP, 42–54%), which was absent from the native grassland samples, the tame grassland samples, and two cropland samples (Supplementary Table S2). Additionally, small amounts of P (<17%, Supplementary Table S2) in the forms of dibasic calcium phosphate (DCP), monobasic calcium phosphate (MCP), hydroxyapatite (HAP), or FePO_4_ were also fitted in some samples. This indicated that these P species may be present in the soils, but there were no significant differences of these P species among land uses types (**Table 3**).

**Table 3:** Phosphorus K-edge XANES fitting results showing the relative percent of each phosphate species^a^ in the studied soils under different land uses (means ± standard errors, *n* = 4)^b^.

Land uses	IHP	TCP	DCP	MCP	HAP	FePO_4_
	
				%		
Roadside	50 ± 3 b	47 ± 3 a	0 a	0 a	3 ± 3 a	0 a
Native grasslands	92 ± 5 a	0 b	6 ± 3 a	0 a	0 a	2 ± 2 a
Tame grasslands	82 ± 4 a	3 ± 3 b	2 ± 2 a	2 ± 2 a	7 ± 4 a	3 ± 3 a
Annual Crop	67 ± 17 ab	24 ± 19 ab	2 ± 2 a	0 a	4 ± 4 a	3 ± 3 a


*^*a*^IHP, inositol hexakisphosphate; TCP, tricalcium phosphate; DCP, dibasic calcium phosphate; MCP, monobasic calcium phosphate; HAP, hydroxyapatite. ^*b*^Values in each column followed by the same lowercase letters are not significantly different according to LSD (P < 0.05).*

### Metagenomic Analysis

Land use had a strong effect on the taxonomic composition of the soil microbial community. Although the archaeal community did not differ, the bacterial and fungal communities significantly differed among the land use types (Supplementary Table S7). Tame and native grassland soils harbored similar bacterial and fungal communities, whereas annual cropland and roadsides clustered separately in the principle coordinate analysis (PCoA, Supplementary Figure S6), indicating they had distinct communities.

**FIGURE 3:**
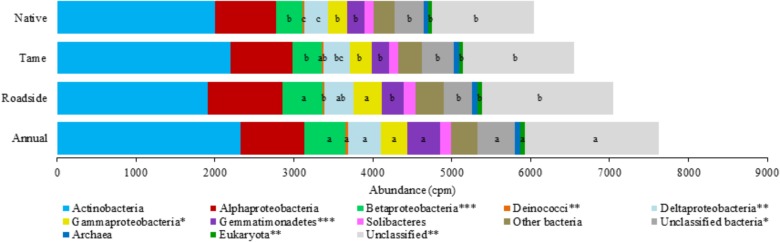
Taxonomic classification and relative abundance (copies per million reads) of the phosphorus cycling genes among the four land use types. Mean values (copies per million reads) within a taxonomic classification that have a different letter indicate a significant difference between land use types (*P* < 0.05). An asterisk (^∗^) indicates a significant land use type effect at *P* < 0.05 (^∗∗^*P* < 0.01 and ^∗∗∗^*P* < 0.001).

The microbial genes coding for various P cycling processes were broadly grouped into six functional categories based on Bergkemper et al. (2016), and these included phosphoesterase, phytase, phosphonate degradation, inorganic phosphate solubilizing, P transporter, and regulation of phosphate starvation genes (**Table 4**). Taxonomic classification of the P cycling genes revealed that the majority of the reads (76.1%) were assigned or classified as bacteria, 0.9% archaea, 0.8% eukaryota, and the remaining 22.1% of the reads were unclassified (**Figure 3**). Actinobacteria and Alphaproteobacteria were the most abundant bacterial classes but did not significantly differ among the land use types. Significant differences were observed for the Betaproteobacteria, Deinococci, Deltaproteobacteria, Gammaproteobacteria, Gemmatimonadetes, and Eukaryota, all of which were the most abundant in annual cropland soils and least abundant in native grassland soils (**Figure 3**). Overall, annual cropland soils had the highest number of reads assigned to P cycling coding genes; native grassland soils had the lowest.

**Table 4:** Abundance of microbial genes associated with phosphorus cycling in soil.

Functional group		Gene	KEGG orthology	Native	Tame	Roadside	Annual
Phosphoesterase genes	Acid phosphatase		K01078	161.39	190.80	155.67	178.88
	Acid phosphatase	phoN	K09474^∗∗∗^	32.51 b	35.70 b	48.10 a	52.06 a
	Acid phosphatase	aphaA	K03788	0.32	0.44	0.35	0.27
	Alkaline phosphatase	phoA	K01077^∗^	36.35 b	41.98 ab	48.40 a	40.33 b
	Alkaline phosphatase	phoX	K01077	0.05	0.00	0.12	0.08
	Alkaline phosphatase	phoD	K01113	411.74	430.55	467.28	505.17
	GP phosphodiesterase	ugpQ	K01126^∗^	323.30 b	354.98 ab	360.65 ab	401.54 a
	Phosphotriesterase		K07048	100.08	105.33	99.03	119.70
Phytase genes	3-Phytase		K01083	77.96	78.82	97.27	116.39
	4-Phytase	appA	K01093^∗^	1.94 a	1.54 ab	2.13 a	1.09 b
Phosphonate degradation genes	C-P lyase multienzyme complex	phnF	K02043^∗∗^	6.30b	6.25b	7.81a	4.89c
	C-P lyase multienzyme complex	phnG	K06166	4.18	4.19	4.77	3.07
	C-P lyase multienzyme complex	phnH	K06165^∗^	5.14 a	5.39 a	5.62 a	4.03 b
	C-P lyase multienzyme complex	phnI	K06164^∗^	9.61 b	9.03 b	13.18 a	9.49 b
	C-P lyase multienzyme complex	phnJ	K06163^∗^	8.14 b	7.86 b	11.38 a	7.89 b
	C-P lyase multienzyme complex	phnK	K05781^∗^	7.02 ab	6.43 b	8.82 a	6.32 b
	C-P lyase multienzyme complex	phnL	K05780^∗∗^	7.17 b	6.39 b	9.08 a	6.16 b
	C-P lyase multienzyme complex	phnM	K06162^∗^	18.77 b	19.94 ab	23.27 a	15.82 b
	C-P lyase multienzyme complex	phnN	K05774^∗∗^	6.89 b	7.46 ab	8.95 a	4.92 c
	C-P lyase multienzyme complex	phnO	K09994^∗^	26.77 ab	29.78 a	22.06 b	30.37 a
	C-P lyase multienzyme complex	phnP	K06167	60.08	63.01	67.10	73.69
	AEP-Pyruvate transaminase	phnW	K03430^∗∗∗^	22.15 b	22.73 b	31.96 a	32.54 a
	Phosphonatase	phnX	K05306	3.76	4.18	4.31	4.75
	Phosphonoacetate hydrolase	phnA	K06193^∗∗^	2.09 b	2.11 b	3.83 a	1.49 b
Inorganic phosphate solubilizing genes	Inorganic pyrophosphatase	ppa	K01507^∗∗^	127.67 c	142.46 bc	144.40 ab	159.73 a
	Exopolyphosphatase	ppx	K01524^∗^	377.44 b	399.73 ab	395.58 b	443.40 a
	Polyphosphate kinase	ppk	K00937^∗^	733.09 b	814.99 ab	809.87 ab	856.84 a
	PQQGDH	gcd	K00117^∗∗^	762.19 b	811.18 b	1206.06 a	1142.91 a
Phosphorus transporter genes	Phosphate inorganic transporter	pit	K03306	266.88	303.67	268.24	280.36
	Low-affinity inorganic phosphate transporter	pitA	K16322^∗^	0.28 ab	0.12 b	0.48 a	0.14 b
	Phosphate-specific transport system subunit	pstA	K02038^∗^	211.81 b	224.38 b	232.04 ab	257.92 a
	Phosphate-specific transport system subunit	pstB	K02036^∗^	199.29 b	218.36 b	221.83 b	252.92 a
	Phosphate-specific transport system subunit	pstC	K02037^∗^	236.61 b	255.90 ab	254.61 ab	286.45 a
	Phosphate-specific transport system subunit	pstS	K02040^∗∗^	298.59 b	325.53 b	332.87 b	375.87 a
	Phosphonate transporter subunit	phnC	K02041^∗^	46.32 b	48.50 b	54.06 ab	62.21 a
	Phosphonate transporter subunit	phnD	K02044^∗^	87.21 b	93.51 b	105.91 ab	128.44 a
	Phosphonate transporter subunit	phnE	K02042^∗^	46.41 b	46.12 b	63.65 a	64.02 a
	Glycerol-3-phosphate transporter subunit	ugpA	K05814^∗^	38.74 c	42.49 bc	51.72 a	49.14 ab
	Glycerol-3-phosphate transporter subunit	ugpB	K05813^∗∗^	74.76 b	78.02 b	93.49 a	95.93 a
	Glycerol-3-phosphate transporter subunit	ugpC	K05816^∗∗∗^	36.76 b	37.08 b	44.76 a	42.56 a
	Glycerol-3-phosphate transporter subunit	ugpE	K05815^∗∗^	38.10 b	40.95 b	48.95 a	49.53 a
Regulation of phosphate starvation inducible genes	Phosphate regulon response regulator	phoB	K07657^∗∗^	215.10 b	229.43 b	237.40 b	278.42 a
	Phosphate regulon sensor histidine kinase	phoR	K07636^∗∗^	718.51 b	799.38 b	768.48 b	943.60 a
	PhoR/PhoB inhibitor protein	phoU	K02039^∗^	188.29 b	204.46 b	208.66 ab	237.55 a


*Mean values (n = 4) represent counts per million of mapped reads for each land use type. Values within a row that have different letters indicate a significant difference between land use types (P < 0.05).^∗^P < 0.05, ^∗∗^P < 0.01, ^∗∗∗^P < 0.001.*

The permanova results showed a significant difference in the composition of the P cycling bacterial communities and composition of the P cycling functional genes among the four land use types (Supplementary Table S7). The PCoA (Supplementary Figure S7A) revealed a similar clustering of the P cycling bacterial communities compared to the total bacterial community (which included taxa that were not linked to P cycling functional genes) among the land use types, with roadside communities being the most distinct and dissimilar to native and tame grassland communities. However, PCoA of the P cycling gene composition revealed that native grassland and annual cropland soils had the most dissimilar P cycling gene compositions, with roadside and tame grassland soils being intermediary (Supplementary Figure S7B).

The abundance of most P cycling genes significantly differed among the land use types (**Table 4**). Genes coding for phosphoesterase enzymes were the most abundant in annual cropland and roadside soils, and least abundant in native grassland soils. Alkaline phosphatase (*phoD*, *phoA*, *phoX*) and glycerophosphoryl diester phosphodiesterase (*upgQ*) were the most abundant phosphoesterase enzyme coding genes for all land use types, possibly indicating that these enzymes have a higher capacity for P mineralization in the soils from this region compared to acid phosphatase and phytase enzymes. Roadside soils had the highest abundance of genes coding for phosphonate degradation enzymes, and this was particularly evident of genes coding for enzymes involved in the carbon-phosphorus (C-P) lyase core complex (*phnG*, *phnH*, *phnI*, *phnJ*, *phnM*). Genes coding for inorganic phosphate solubilizing enzymes (*ppa*, *ppx*, *ppk*, *gcd*) were the most abundant group of P cycling genes for all land use types. Annual cropland and roadside soils appeared to have the highest inorganic phosphate solubilizing capacity based on the abundance of these genes, and native grassland soils the lowest capacity. Annual cropland soils also had the highest abundance of genes coding for P transporter subunits (phosphate-specific transporter, phosphonate transporter, and glycerol-3-phosphate transporter) and genes regulating phosphate starvation (*phoB*, *phoR*, *phoU*). Native grassland and tame grassland soils exhibited a lower phosphate uptake and regulation capacity based on the lower abundance of these genes.

Supplementary Figure S8 shows the taxonomic composition (at the class level) of each P cycling gene. There are similarities in the taxonomic composition of genes that are functionally related. For example, most genes coding for the C-P lyase multienzyme complex are primarily from Alphaproteobacteria and from Actinobacteria and Betaproteobacteria to a lesser extent. The genes coding for the phosphate-specific transport subunit (*pstA*, *pstB*, *pstC*, *pstS*), phosphonate transporter subunit (*phnC*, *phnD*, *phnE*), and glycerol-3-phosphate transporter subunit (*ugpA*, *ugpB*, *ugpC*, *ugpE*) each had similar taxonomic compositions among the genes that make up their respective subunits. In contrast, other genes coding for similar functions such as alkaline phosphatase (*phoA*, *phoX*, *phoD*) and acid phosphatase (*phoN*, *aphA*, K01078) had higher variability in their respective taxonomic compositions.

### Redundancy Analysis

**FIGURE 4:**
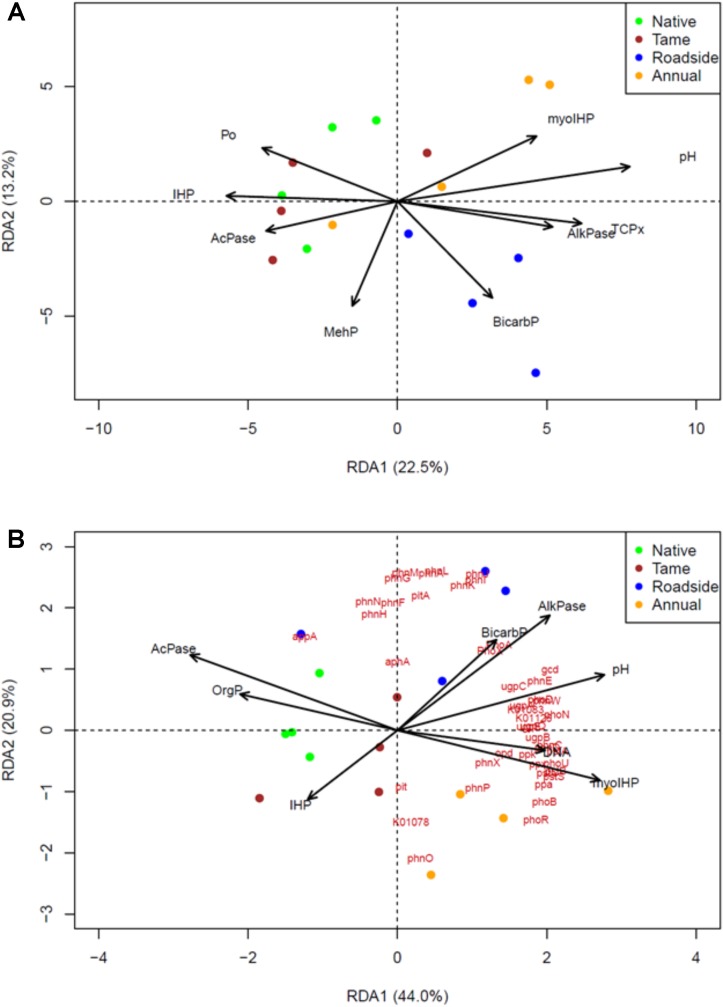
Identification of the P chemistry related drivers of **(A)** the P-cycling soil bacterial community, and **(B)** soil P-cycling functional gene composition among the four land use types based on the metagenomic data sets and redundancy analysis (RDA). A step-wise selection of important predictors of gene and community composition was used in conjunction with the RDA. The biplot arrows represent the P-chemistry related variables and red text in **(B)** represent the P-cycling functional genes or KEGG orthology numbers. Green, brown, blue and yellow colors, respectively, represent native grasslands, tame grasslands, roadsides, and annual croplands.

Redundancy analysis (RDA) was used to identify the most important P chemistry predictors of the P cycling bacterial community composition (**Figure 4A**) and P cycling gene composition (**Figure 4B**). Similar P chemistry predictors were identified for both the bacterial community and gene compositions with a few exceptions. Overall, NMR (*myo*-IHP and IHP), enzyme activity (acid and alkaline phosphatase), Olsen P, and pH were significantly correlated with the composition of both the bacterial and functional gene compositions. In contrast, P-XANES species exhibited a limited relationship as only TCP was identified as a significant predictor of the P cycling bacterial community composition. The P chemistry predictors were able to explain a high proportion of the P cycling bacterial community and functional gene composition as the adjusted *r*^2^-values for each RDA was 0.34 and 0.75, respectively.

## Discussion

The results by the ignition method, NMR, and XANES consistently indicated that native and tame grasslands had higher total P_o_ proportions among the investigated land use types. In grazed grasslands, the majority of the P (∼85%) taken up in the form of orthophosphate by plants is returned to the soil in the form of P_o_ through dung, providing a significant P_o_ stock (Nash et al., 2014). Specifically, differences among land use types were significant for monoesters, principally total IHP and the *neo*-IHP stereoisomer. Higher proportions of monoester P in grasslands than cultivated soils have been reported previously (Condron et al., 1990; Stutter et al., 2015), although these studies did not correct for diester degradation or identify specific P forms. Using an improved method of P-NMR spectra interpretation to identify specific P forms, we found a significantly higher abundance of *neo*-IHP in native grasslands and an unidentified monoester species resolved at 4.9 ppm, which might also be *neo*-IHP (Turner et al., 2012), in tame grasslands compared to croplands. The abundance of *chiro*-IHP was extremely high in the tame pasture soil from one location, but the overall abundance of either of the two configurations of *chiro*-IHP was not significantly different among land use types. The specific origins and function of *chiro*-IHP and *neo*-IHP in soils are unknown, but they are thought to be synthesized by soil microbes (Turner et al., 2002, 2014; Giles et al., 2011). The higher abundance of these stereoisomers and lower ratio of *myo*-IHP:other IHP forms (**Table 2**) in grassland soils indicates that microbes are likely responsible for the higher accumulation of IHP compared to annual cropland and roadside soils.

The higher abundance of *neo*-IHP in grassland soils may be linked to reduced P turnover or cycling in these land use types. For example, Young et al. (2013) reported significantly higher *neo*-IHP concentrations under conditions (i.e., poor drainage) that constrain microbial P cycling. In the present study, native and tame grassland soils had the lowest abundance of genes coding for most P cycling processes (except phosphonate degradation) compared to other land use types, which may be an indicator of reduced P cycling capacity. The accumulation of *neo*-IHP in the grassland soils of this study is consistent with the results of Young et al. (2013), supporting their speculation that this IHP stereoisomer accumulates when microbial P cycling is reduced.

The native grassland soils of this study had a greater abundance of the 4-phytase gene (*appA*) than cropland soils, although there were no significant differences among land use types for the more abundant 3-phytase genes (K01083). Differing from other P_o_ mineralization processes, phytases may not be controlled by the PhoBR two component regulatory system, but likely respond to the presence or absence of phytate in the soil (Lidbury et al., 2016). However, the ability of these genes to degrade other stereoisomers besides phytate (*myo*-IHP) is unknown, and previous research suggests *neo*-, *chiro*-, and *scyllo*-IHP are more resistant to phytase hydrolysis than the *myo*-IHP stereoisomer (Turner et al., 2012). It has been suggested that microbes synthesize these stereoisomers as a potential strategy to preserve phosphorus from competing organisms under limiting P conditions (Turner, 2007). Contrary to the anecdotal evidence that P_o_ mineralization in pastures might be high (Nash et al., 2014), our results demonstrated that the abundance of most P cycling genes was lowest in native grasslands, except for the 4-phytase gene. This suggests that P_o_ turnover in grasslands from this region may not be as active as other more intensive land use types, allowing P_o_ to accumulate or become immobilized in soils via microbial processes. This is particularly relevant in grassland soils with low P_i_ availability (Bünemann et al., 2012). Given the lower degree of disturbance in grasslands as a long-term stable ecosystem (Cade-Menun et al., 2017a), the transformation of P_o_ in grasslands is likely more tightly regulated than arable systems and driven by intrinsic soil-plant-microbial cycling demands for P. It is clear that P_o_ serves as a substantial reserve of P for plant nutrition in grasslands. As such, further investigations linking the supply of orthophosphate from these P_o_ species with plant needs warrants further studies (Nash et al., 2014).

In annual croplands, P_o_ returns to soils are interrupted at crop harvest, which may account for the limited P_o_ accumulation in this study. However, enhanced mineralization of P_o_ by microbe-mediated activities due to low soil Olsen-P cannot be excluded, since annual croplands had the highest total abundance of genes coding for phosphoesterase and phytase enzymes. This contradicts the lower acid phosphatase and phosphodiesterase activity we observed in annual cropland soil despite the higher abundance of genes coding for these enzymes (*phoN*, *ugpQ*) compared to the other land use types. One explanation for this observation may be linked to the pH conditions of the enzyme assays as this can affect the relationships between gene abundance and enzyme activity (Fraser et al., 2017).

The high abundance of P cycling genes suggests a greater capacity for P transformation in cropland soils of this study. This may be a strategy utilized by the soil microbial community to adapt to the high temporal variability in P forms and availability (Hedley et al., 1982; Bainard et al., 2016) associated with the intensive practices of annual crop production (e.g., fertilization, tillage, harvest, weed management and fallow). There was considerable variability in crop management practices among the annual cropland sampling sites, including fertilizer and P-containing herbicide inputs (Cade-Menun et al., 2017a), which may have contributed to the high site variance in P_i_ species and the insignificant differences among the four land use types. Additionally, the relatively higher TCP in the annual croplands than grasslands may arise from tillage, because the surface of annual croplands could be replenished by the deeper soil containing high levels of carbonates after repeated tillage. The higher abundance of P_i_ solubilizing genes in annual cropland soils could indicate a higher capacity for the microbial community to access these P_i_ forms compared to the grasslands. Interestingly, glyphosate was commonly used in three of the four annual cropping sites, but these soils had the lowest abundance of genes coding for the polypeptides that make up the core complex or reaction of the C-P lyase pathway, which is one of the primary pathways for the catabolism of phosphonates (Hove-Jensen et al., 2014). However, all polypeptides involved in the C-P lyase pathway (*phnCDEFGHIJKLMNOP*) are required for the utilization of glyphosate as a phosphate source (Chen et al., 1990; Hove-Jensen et al., 2014), including those that are more abundant in annual cropland soil (phosphonate transporters *phnCDE*, aminoalkylphosphonate N-acetyltransferase *phnO*, and phosphoribosyl cyclic phosphodiesterase *phnP*). Glyphosate has a distinctive peak in P-NMR spectra (Cade-Menun, 2015), which was not detected in the cropland soils, and the concentrations of phosphonates in general were not significantly greater in cropland soils than other land use types in this study. Further investigation is warranted to understand the factors controlling the degradation of agricultural compounds such as glyphosate in the soils of this region.

Annual crop production practices appear to have an impact on the PhoBR two component regulatory system based on the higher abundance of P-starvation-inducible genes compared to the other land use types. This is consistent with the low Olsen *P*-values for the cropland soils. This system regulates several important P cycling processes controlled by genes coding for phosphoesterase, phosphonate degradation, inorganic phosphate solubilizing, and P transporter enzymes (Furtwängler et al., 2010; Lidbury et al., 2016). Bergkemper et al. (2016) suggested that a high abundance of P-starvation-inducible genes can enable microbial communities to utilize alternative P forms under P limiting conditions. This may be more important in annual cropland soils compared to perennial grasslands, because grassland ecosystems likely experience fewer disturbances and fluxes in P availability.

Roadside soils differ from the other land use types because they are disturbed environments that have high variability in terms of soil chemical and physical properties and soil moisture gradients due to runoff from adjacent roads and croplands and nutrient removal via haying and mowing (Dai et al., 2013; Cade-Menun et al., 2017a). Roadside soils distinguished from other land use types in this study based on having the highest Olsen P and TCP concentrations and low P_o_. In a separate study, Cade-Menun et al. (2017a) showed that roadside soils collected from the same sampling locations as the current study at the 0–7.5 cm depth had the greatest percentage of clay, the highest soil pH and higher total C relative to the other land use types. There were generally no differences among land use types at lower depths; thus, sampling at 0–30 cm may have obscured some differences for the current study. Roadsides in Saskatchewan are subjected to snowmelt runoff, in which dissolved reactive P accounted for 97–100% of the dissolved total P loss from croplands and pastures (Cade-Menun et al., 2013), which would account for the high Olsen P concentrations in these roadside soils. These roadside soils would also experience deposition of clays during wind erosion (Cade-Menun et al., 2017a). This, with the neutral pH (7.1) and high NH_4_OAC-extracted Ca are expected to facilitate the formation of TCP in these roadside soils (Sato et al., 2005). From the microbial perspective, the relatively high abundance of P cycling functional genes along with high phosphatase activity and low abundance of P_o_ indicate that microbe-mediated P dynamics in roadsides are active but highly variable.

To the best of our knowledge, this is the first study to combine state-of-the art spectroscopic methods for P chemistry with shotgun metagenomics to provide an in depth evaluation of the dominant mechanisms involved in P cycling in soils with different land uses. Grasslands had high abundances of monoesters, principally IHP stereoisomers, and high acid phosphomonoesterase activities but lower abundance of genes coding for P cycling processes. In particular, the significantly higher proportion of *neo*-IHP in the native grasslands than in croplands confirms the important role of microbes in P_o_ transformation in grassland soils. In contrast, croplands showed the largest variance of P speciation among the land use types, illustrating the crucial role of specific field management practices within croplands. Furthermore, the conversion of native grassland to annual crop production appears to increase the abundance of P-cycling genes, which may be required in soils that are under intensive management practices. Future studies are warranted to design tailored agronomic practices that directly facilitate functional genes and microbial communities for certain P cycling processes (e.g., P_o_ mineralization) to optimize P-use efficiency. Roadside soils had the highest Olsen-P due to inputs from erosion and runoff, and had high proportions of TCP, reflecting clay inputs, neutral pH and high exchangeable Ca concentrations. The RDA results demonstrated that IHP by NMR, enzyme activity, Olsen-P, and pH were important P chemistry predictors of the P cycling bacterial community composition and functional gene composition.

## Author Contributions

LB, BC-M, MS, KL, and CH designed and conducted the study. The NMR analysis was performed by CL and interpreted by BC-M. JL and JY conducted the synchrotron experiments. JL, JY, and YH analyzed the data. LB and JT performed the soil microbial and bioinformatic analyses. JL, LB, and BC-M wrote the manuscript with inputs from all authors. All authors read and approved the manuscript.

## Conflict of Interest Statement

The authors declare that the research was conducted in the absence of any commercial or financial relationships that could be construed as a potential conflict of interest.

## Footnotes

1 http://duk.sourceforge.net/

2 http://bio-bwa.sourceforge.net
