# Supplementary material for: Long-Term Land Use Affects Phosphorus Speciation and the Composition of Phosphorus Cycling Genes in Agricultural Soils
Source: Front Microbiol. 2018 Jul 20;9:1643. doi: 10.3389/fmicb.2018.01643 (PMC6065304; doi:10.3389/fmicb.2018.01643)
Supplement: Supplementary file 1 [file Presentation_1.pdf]

Supplementary Material

Long-term Land Use Affects Phosphorus Speciation and the Composition of Phosphorus Cycling Genes in Agricultural Soils

Jin Liu[1, 2], Barbara J. Cade-Menun[3], Jianjun Yang[4], Yongfeng Hu[5], Corey W. Liu[6], Julien Tremblay[7], Kerry LaForge[3], Michael Schellenberg[3], Chantal Hamel[3], Luke Bainard[3]*

[1]College of Agronomy and Biotechnology, China Agricultural University, Beijing, China
[2]Visiting Scientist, Agriculture and Agri-Food Canada, Swift Current Research and Development Centre, Swift Current, SK, Canada
[3]Swift Current Research and Development Centre, Agriculture and Agri-Food Canada, Swift Current, SK, Canada
[4]Institute of Environment and Sustainable Development in Agriculture, Chinese Academy of Agricultural Sciences, Beijing, China
[5]Canadian Light Source, University of Saskatchewan, Saskatoon, SK, Canada
[6]Stanford Magnetic Resonance Laboratory, Stanford University School of Medicine & ChEM-H-Stanford University, Stanford, CA, USA
[7]Energy, Mining and Environment, National Research Council of Canada, Montreal, QC, Canada

*** Correspondence:**
Luke D. Bainard, Agriculture and Agri-Food Canada, Swift Current Research and Development Centre, Box 1030, Swift Current, SK, Canada; luke.bainard@agr.gc.ca; telephone 306-770-4497.

1 Supplementary Figures and Tables

1.1 Supplementary Figures

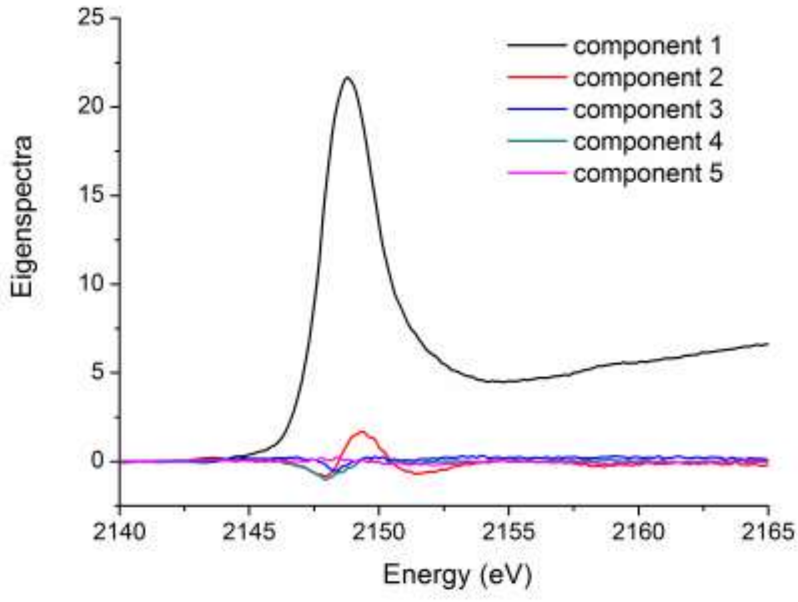

Figure S1. Eigenspectra of the first five components obtained from principal component analysis (PCA) on P K-edge XANES spectra of the studied soil samples.

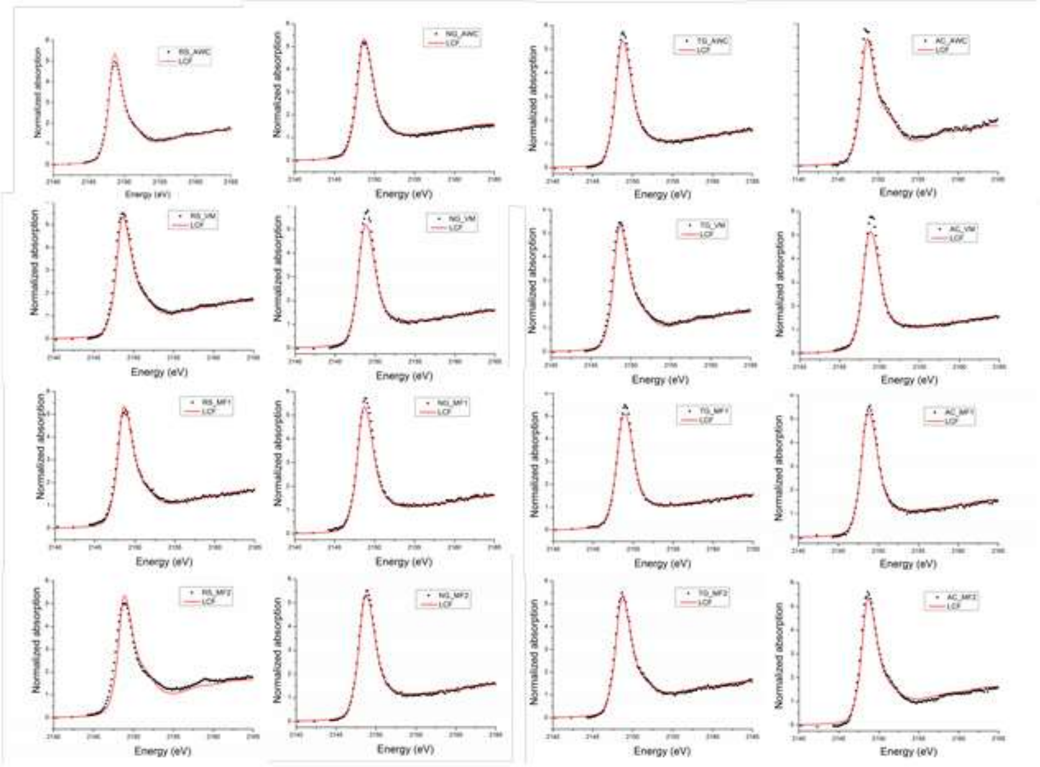

Figure S2. Linear combination fitting of P K-edge XANES spectra of the investigated soils collected at four locations of Val Marie (VM), Auvergne Wise Creek (AWC), Masefield1 (MF1) and Masefield2 (MF2) under different land uses including roadside soils (RS), native grasslands (NG), tame grasslands (TG) and annual croplands (AC).

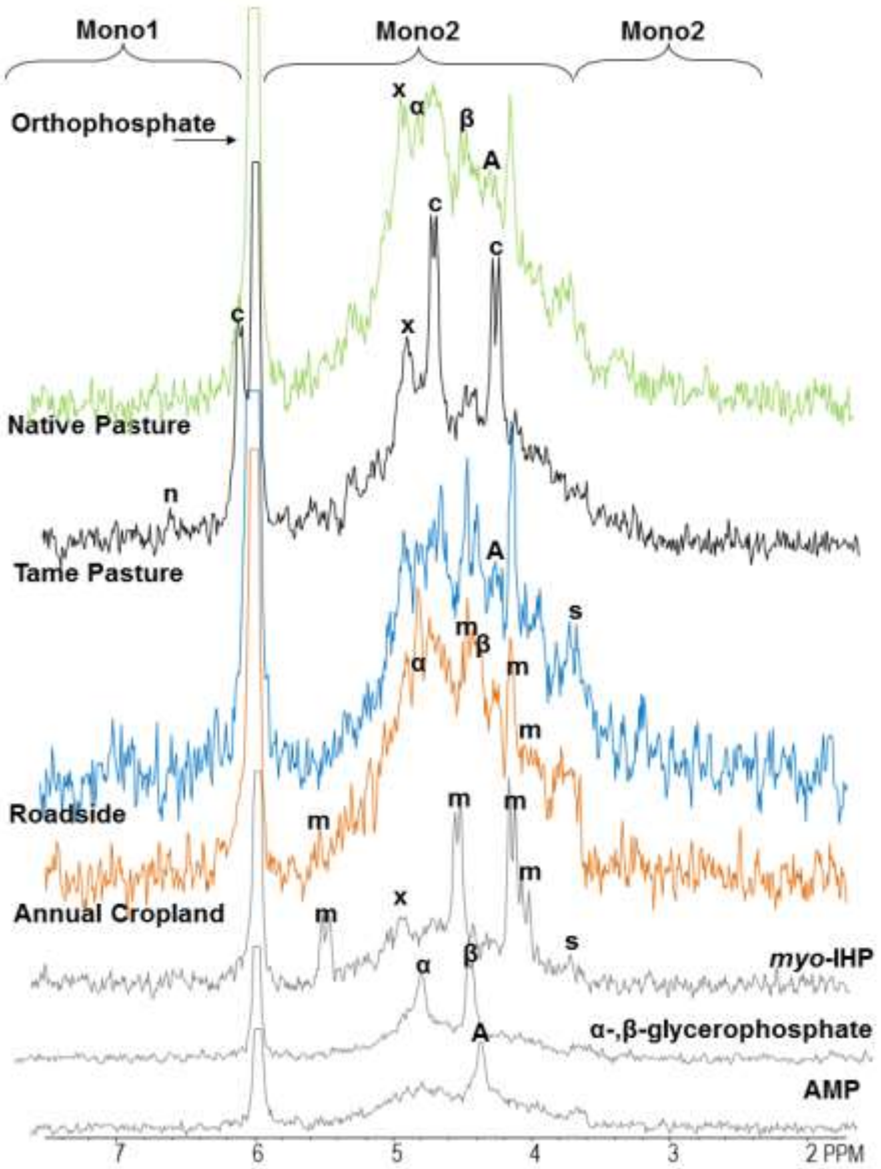

Figure S3. Spectra (P-NMR) zoomed in to show details of the orthophosphate monoester region. Spectra were processed with 2 Hz line-broadening, and are plotted to full height and scaled to the height of the tallest peak in the Mono2 region. Note that the orthophosphate peak is truncated. The colored spectra show the samples for each land use type from the Masefield 1 location; the grey spectra are samples from this project into which P forms, indicated for each spectrum, were added to help with peak identification. Mono1, Mono2, Mono3, general regions of the orthophosphate monoester region; α, α-glycerophosphate; β, β-glycerophosphate; A, adenosine monophosphate (AMP); c, *chiro*-inositol hexakisphosphate (IHP), 4 axial 2 equatorial conformation; m, *myo*-IHP; n, *neo*-IHP; s, *scyllo*-IHP; x, unknown P compound. Note that not all peaks in all spectra are labelled, even if the peaks are present.

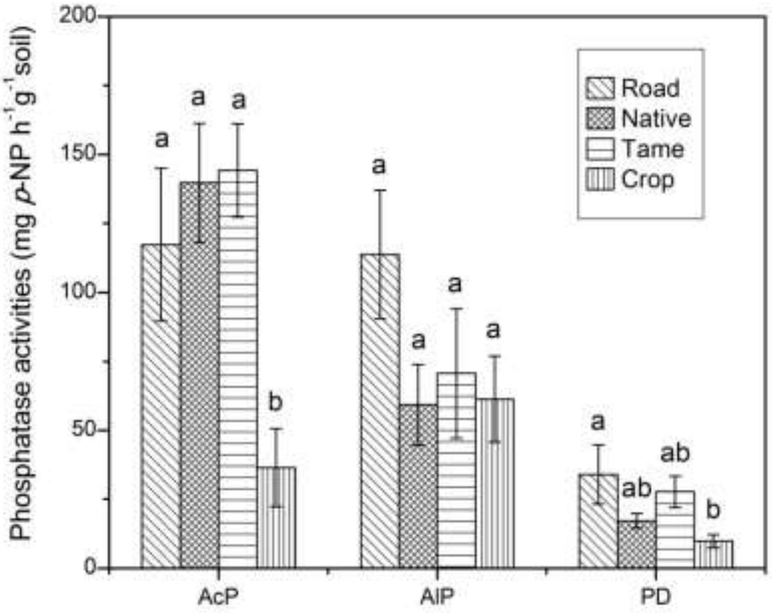

Figure S4. Phosphatase activities of the studied soils under different land use. AcP, acid phosphomonoesterase; AlP, alkaline phosphomonoesterase; PD, phosphodiesterase. Significant letters indicate significant differences in enzyme activities among land use types for each enzyme.

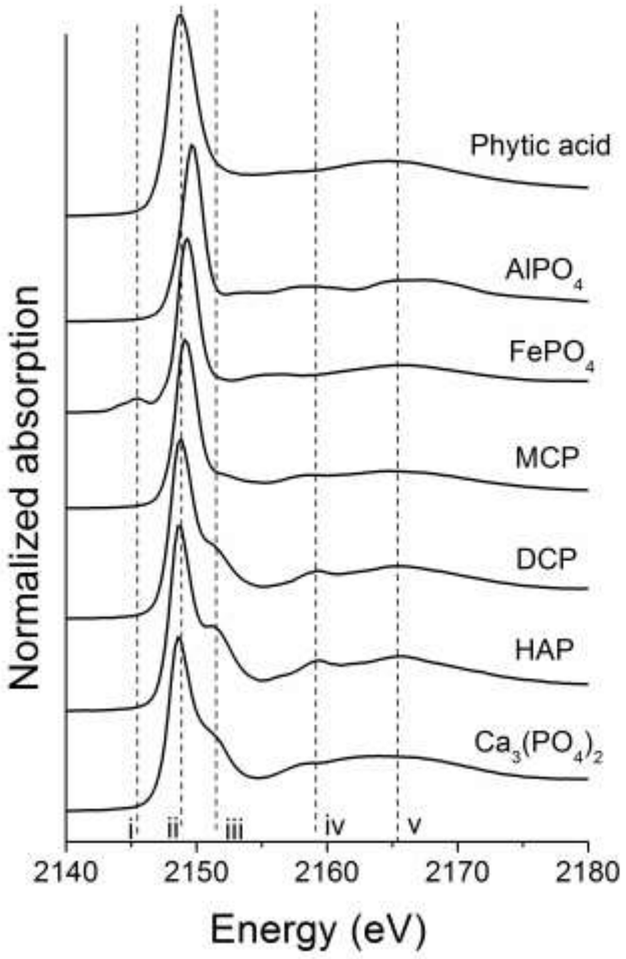

Figure S5. Phosphorus K-edge XANES spectra of P references. MCP, $Ca(H_2PO_4)_2$; DCP, $CaHPO_4$; HAP, hydroxyapatite.

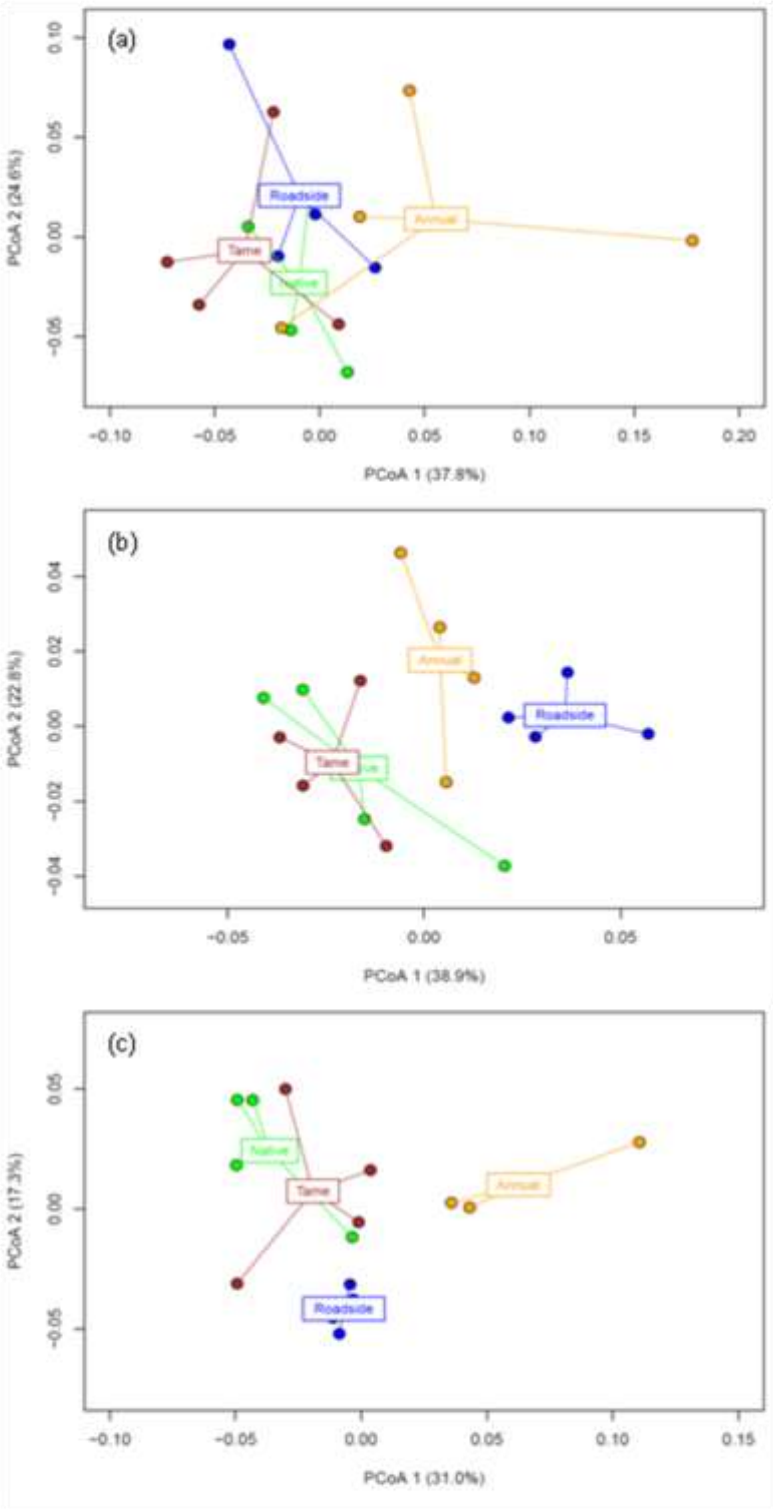

Figure S6. Composition of the soil (a) archaeal, (b) bacterial, and (c) fungal communities among the four land use types based on the metagenomic data sets and principle coordinate analysis (PCoA) of Bray-Curtis distances. Green, brown, blue and yellow colours respectively represent native grasslands, tame grasslands, roadsides, and annual croplands.

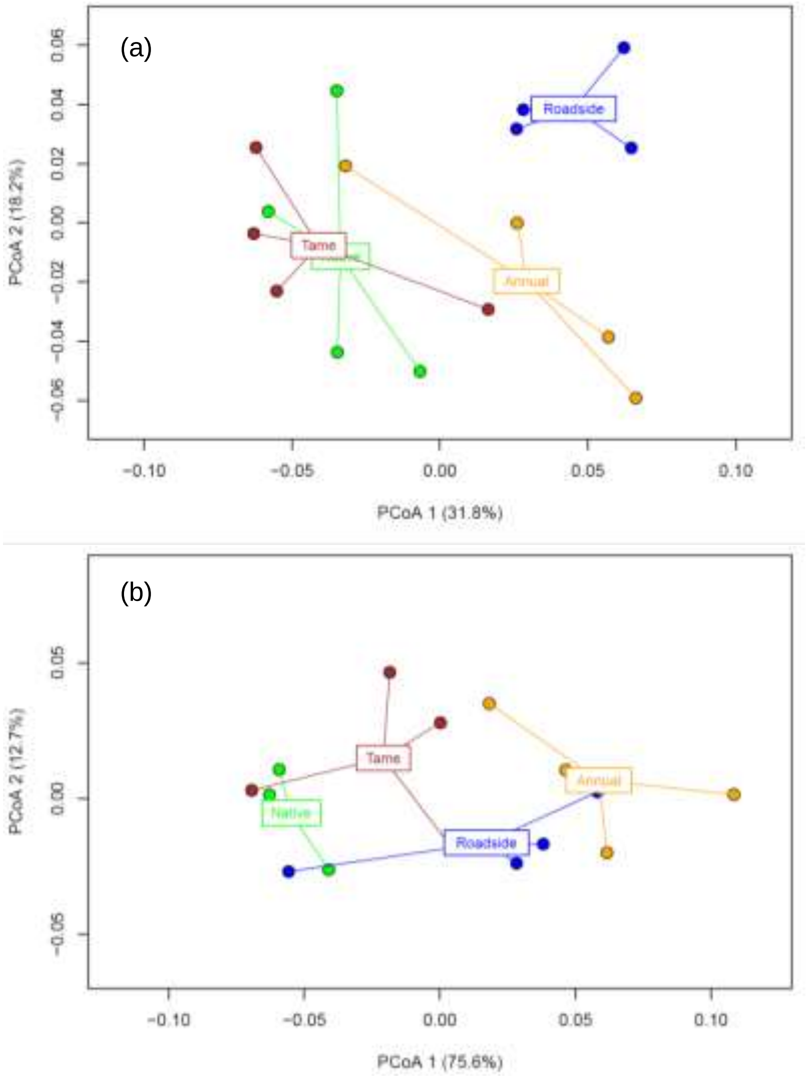

Figure S7. Composition of the (a) P cycling bacterial community and (b) P cycling functional genes among the four land use types based on the metagenomic data sets and principle coordinate analysis (PCoA) of Bray-Curtis distances. Green, brown, blue and yellow colours respectively represent native grasslands, tame grasslands, roadsides, and annual croplands.

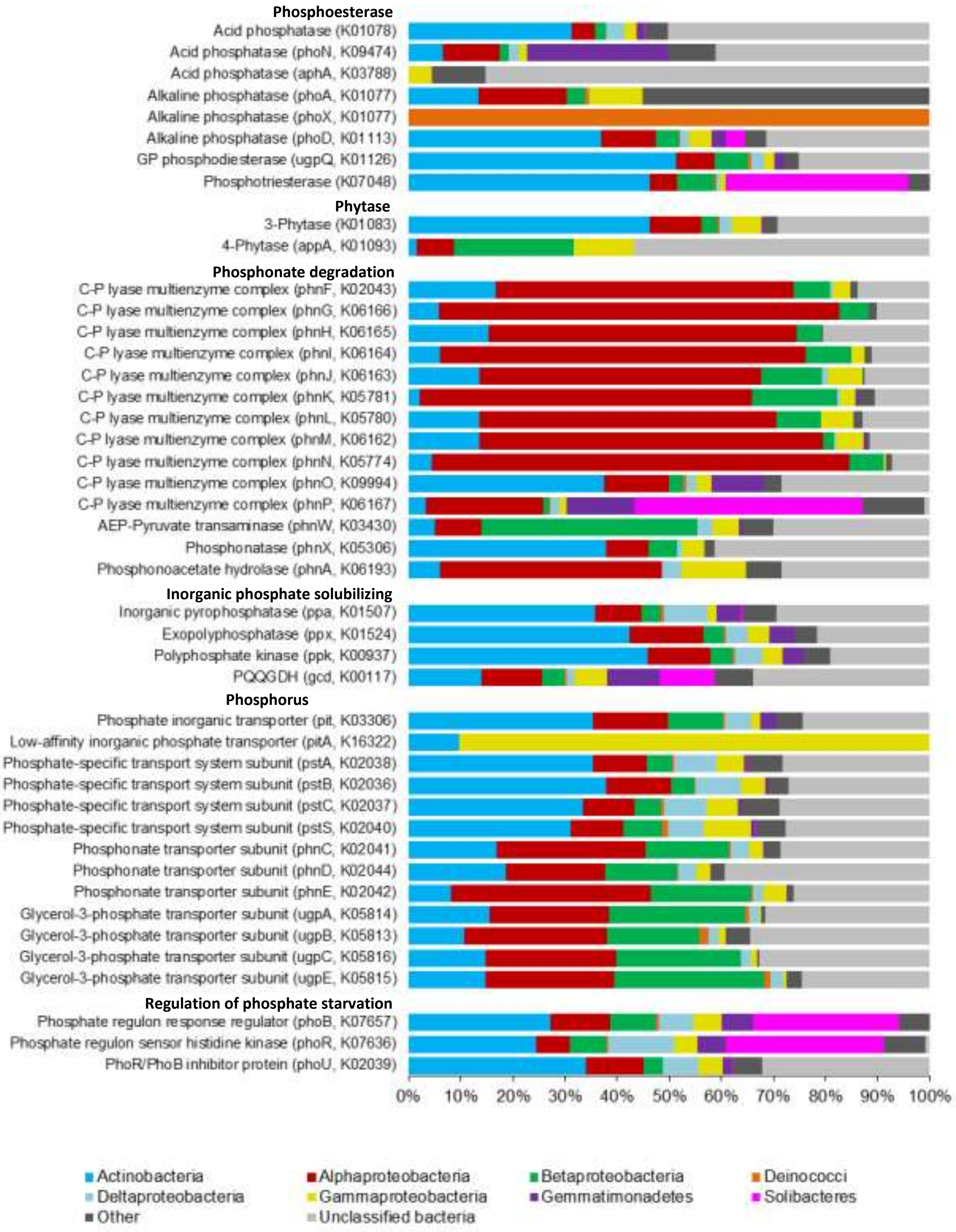

Figure S8. Taxonomic composition of phosphorus cycling functional genes based on the metagenomic data sets. Gene functions were assigned by matching predicted genes of the co-assembled metagenome against the KEGG genes database.

1.2 Supplementary Tables

Table S1. Results of the first five components obtained from principle component analysis (PCA) on P K-edge XANES spectra of the studied soil samples.

Components	Eigenvalue	Variation	Accumulated variation	IND
Component 1	156.9	0.840	0.840	0.0120
Component 2	6.862	0.036	0.877	0.0108
Component 3	5.170	0.027	0.904	0.0098
Component 4	3.810	0.020	0.925	0.0092
Component 5	2.581	0.013	0.939	0.0095

Table S2. Phosphorus K-edge XANES fitting results showing the relative percent of each phosphate species in the studied soil samples under different land uses.

Land use	location[b]	x^2	R factor	Percentages of targeted components %					
				IHP	TCP	DCP	MCP	HAP	$FePO_4$
	AWC	2.068	0.004	46	54	-			
Roadsides	VM	4.850	0.008	51	49				
	MF1	1.344	0.002	58	42	-			
	MF2	8.131	0.014	43	45	-	-	12	
	AWC	1.747	0.003	100	-	-	-	-	-
Native grasslands	VM	3.071	0.005	78	-	12	-	-	10
	MF1	2.316	0.004	88	-	12	-	-	-
	MF2	0.848	0.002	100	-	-	-	-	-
	AWC	2.045	0.004	90	-	10	-	-	-
Tame grasslands	VM	4.421	0.007	88	-	-	-	12	-
	MF1	1.085	0.002	79	-	-	10	-	11
	MF2	1.348	0.003	72	11	-	-	17	
	AWC	5.993	0.009	20	80	-			
Annual croplands	VM	3.261	0.006	78	-	10	-	-	12
	MF1	1.162	0.002	100	-	-	-	-	-
	MF2	3.485	0.006	68	15	-	-	17	-

[a] IHP, inositol hexakisphosphate; TCP, tricalcium phosphate; DCP, dibasic calcium phosphate; MCP, calcium phosphate monobasic; HAP, hydroxyapatite

[b] four locations including Val Marie (VM), Auvergne Wise Creek (AWC), , Masefield1 (MF1) and Masefield2 (MF2).

Table S3. Read count summaries and mapping statistics

Land use	Location	Raw Fragments	Surviving Fragments	Surviving Fragments%	Surviving Single	Total Reads QCed	Mapped	Mapped%	Properly Paired	Properly Paired%
Annual cropland	AWC	51,310,853	49,262,611	96%	1,669,388	98,518,012	66,726,659	67%	49,529,234	50%
Tame grassland	AWC	46,355,362	44,191,424	95%	1,796,591	88,373,630	63,590,571	71%	46,422,046	52%
Native grassland	AWC	36,797,199	35,215,433	95%	1,278,149	70,422,390	48,913,068	69%	36,473,824	51%
Roadside	AWC	63,139,252	60,266,333	95%	2,466,359	120,528,294	77,642,280	64%	56,779,294	47%
Annual cropland	MF1	59,445,678	57,446,871	96%	1,612,013	114,890,574	77,183,890	67%	58,102,852	50%
Tame grassland	MF1	48,145,693	45,895,219	95%	1,894,269	91,783,844	58,517,926	63%	43,406,616	47%
Native grassland	MF1	39,124,209	37,620,062	96%	1,199,183	75,232,162	52,344,434	69%	39,152,028	52%
Roadside	MF1	52,895,185	50,856,159	96%	1,657,408	101,703,394	65,356,276	64%	48,097,478	47%
Annual cropland	MF2	62,255,615	59,976,986	96%	1,791,252	119,943,414	80,797,617	67%	60,906,162	50%
Tame grassland	MF2	36,884,307	35,640,735	96%	1,019,928	71,277,706	50,735,826	71%	37,323,674	52%
Native grassland	MF2	38,000,832	36,713,862	96%	1,051,290	73,422,692	51,901,604	70%	38,541,106	52%
Roadside	MF2	38,723,748	36,453,806	94%	1,957,838	72,900,776	46,276,101	63%	34,530,348	47%
Annual cropland	VM	52,370,549	50,326,666	96%	1,644,656	100,645,130	66,962,572	66%	51,231,132	50%
Tame grassland	VM	42,928,758	41,665,263	97%	975,600	83,322,894	58,604,855	70%	43,775,838	52%
Native grassland	VM	37,423,654	35,833,575	95%	1,267,091	71,658,448	43,916,950	61%	32,503,230	45%
Roadside	VM	52,019,703	49,778,670	95%	1,851,108	99,544,846	63,011,239	63%	47,616,400	47%
Total fragments		909,483,858								
Total reads		1818967716								
Total bases		2.72845E+11								
Total bases (Gb)		272.8451574								

Table S4. Assembly statistics

Assembly stats	Value
contigs greater than 0.5kb	9,235,997
contigs greater than 1kb	1,815,803
contigs greater than 2kb	327,119
contigs greater than 5kb	29,580
contigs greater than 10kb	3,675
contigs greater than 20kb	292
contigs greater than 40kb	17
contigs greater than 80kb	1
contigs greater than 160kb	0
contigs greater than 320kb	0
contigs greater than 640kb	0
contigs greater than 1Mb	0
contigs greater than 2Mb	0
contigs greater than 4Mb	0
contigs greater than 6Mb	0
contigs greater than 8Mb	0
contigs greater than 10Mb	0
Total contigs	9,235,997
Total bases in contigs (bp)	7,905,564,407
Minimum contigs length (bp)	500
Maximum contigs length (bp)	83,187
GC content (%)	64.91
N25 - 25% of total sequence length is contained in the 184,805 sequence(s) having a length >= 1,322 bp	
N50 - 50% of total sequence length is contained in the 517,365 sequence(s) having a length >= 826 bp	
N75 - 75% of total sequence length is contained in the 950,657 sequence(s) having a length >= 620 bp	
N90 - 90% of total sequence length is contained in the 1,251,656 sequence(s) having a length >= 543 bp	

Table S5. Chemical shifts of peaks detected in ^{31}P-NMR spectra. Peaks were present in at least six of the 16 analyzed samples.

P Form or Compound Class	Chemical Shift (ppm)
Inorganic P	
Orthophosphate	6.00 ± 0.00
Pyrophosphate	-4.13 ± 0.05
Polyphosphates	-4.01 ± 0.02, -4.86 ± 0.50, -6.43 ± 0.87, -8.90 ± 0.52, -11.17 ± 0.58, -12.89 ± 0.62, -14.52 ± 0.79, -16.09± 0.57, -19.31 ± 0.55, -20.88 ± 0.78, -22.75 ± 0.72, -25.53 ± 0.68, -28.82 ± 1.23,
Organic P	
Phosphonates	29.87 ± 0.47, 28.53 ± 0.87, 26.04 ± 0.85, 23.48 ± 0.77, 20.95 ± 0.86, 19.10 ± 0.51, 17.24 ± 0.38, 15.75 ± 0.72, 14.11± 0.73, 10.93 ± 0.99, 7.23 ± 0.18
Orthophosphate Monoesters	
myo- IHP	5.54 ± 0.02, 4.57 ± 0.02, 4.11 ± 0.03, 4.05 ± 0.03
scyllo- IHP	3.74 ± 0.03
neo- IHP 4e/2a	6.36 ± 0.03, 4.24 ± 0.02
D-*chiro*-IHP 4e/2a	6.54 ± 0.05, 5.34 ± 0.02, 3.99± 0.03
D-*chiro*-IHP 4a/2e	6.14 ± 0.02, 4.74 ± 0.02, 4.34 ± 0.03
Choline phosphate	3.86 ± 0.04
Glucose 6-phosphate	5.25 ± 0.01
Glucose 1-phosphate	3.35 ± 0.02
Unknown	4.92 ± 0.04
Mono 1	6.81 ± 0.07, 6.70 ± 0.05, 6.44 ± 0.04, 6.25 ± 0.03
Mono 2	5.85 ± 0.03, 5.70 ± 0.06, 5.58 ± 0.05, 5.42 ± 0.06, 5.23 ± 0.08, 4.75 ± 0.05, 4.65 ± 0.03
Mono 3	3.65 ± 0.03, 3.54 ± 0.03, 3.44± 0.03, 3.26 ± 0.06, 3.11 ± 0.07, 2.94 ± 0.11, 2.70 ± 0.04
Orthophosphate Diesters	
Other Diester 1	2.32 ± 0.12, 1.99 ± 0.11, 1.52 ± 0.17, 1.23 ± 0.26, 0.88 ± 0.24, 0.47 ± 0.19, 0.21 ± 0.12, -0.15 ± 0.18, -0.45 ± 0.12
DNA	-0.74 ± 0.05, -0.89 ± 0.06
Other Diester 2	-1.19 ± 0.10, -1.59 ± 0.22, -1.98 ± 0.18, -2.60 ± 0.35, -3.18 ± 0.17, -3.59 ± 0.20
α-glycerophosphate	4.84 ± 0.02
ß-glycerophosphate	4.52 ± 0.01
Mononucleotides	4.47 ± 0.02, 4.42 ± 0.02, 4.36 ± 0.04, 4.26 ± 0.03, 4.15 ± 0.02

Phosphorus forms: IHP, inositol hexaphosphates; *myo*-, *scyllo*-, *neo*-, *chiro*-IHP, stereoisomers of IHP; Mono1, Mono2, Mono3, peaks not specifically identified in three regions of the orthophosphate monoesters; OthDi1, OthDi2, peaks not specifically identified in the orthophosphate diester region.. 4e/2a, 4 equatorial, 2 axial configuration; 4a/2e, 4 axial, 2 equatorial configuration. Unknown, a peak that was identified in spectra, but not specifically identified with respect to P form; the chemical shift is consistent with the 4a/2e configuration of *neo*- IHP, but this identification could not be confirmed with spiking.

Table S6. Phosphorus forms[a] determined by ^{31}P Nuclear Magnetic Resonance for the studied soil samples under different land uses (means ± standard errors, n=4)[b].

Land uses	OrthoP	Pyro	Poly	Phos	myo-IHP	scy-IHP	neo-IHP	chiro-IHP1	chiro-IHP2	4.9ppm	α-glyc	β-glyc
						%						
Roadside	43.1±6.0 a	1.0±0.2 a	2.2±0.5 a	1.4±0.3 a	4.1±0.4 a	2.8±0.4 a	0.7±0 ab	1.5±0.3 a	1.8±0.2 a	2.9±0.5 b	1.1±0.2 a	2.3±0.3 a
Native grassland	27.8±2.4 b	1.3±0.4 a	2.4±0.1 a	1.6±0.2 a	4.1±0.4 a	2.6±0.2 a	1.1±0.2 a	2.0±0.1 a	3.9±1.0 a	4.6±1.0 ab	1.5±0 a	2.4±0.2 a
Tame grassland	27.8±2.9 b	0.7±0.1 a	2.4±0.1 a	1.8±0.4 a	4.2±0.4 a	2.9±0.4 a	0.7±0 ab	2.1±0.2 a	7.3±5.0 a	7.6±1.0 a	1.3±0.4 a	2.5±0.6 a
Annual Crop	38.4±1.9 ab	0.7±0.3 a	2.2±0.4 a	1.3±0.2 a	4.7±0.6 a	2.6±0.3 a	0.6±0.2 b	2.0±0.3 a	2.9±0.5 a	3.9±1.2 b	1.4±0.2 a	2.6±0.4 a

Land uses	Nucl	Pchol	G1P	G6P	Mono1	Mono2	Mono3	DNA	OthDi1	OthDi2
						%				
Roadside	12.3±1.4 a	1.4±0 a	0.7±0 a	0.7±0 a	0.7±0 a	12.0±2.0 a	3.3±0.9 a	0.8±0.3 a	2.1±1.4 a	0.8±0.2 a
Native grassland	16.1±1.3 a	1.5±0 a	0.7±0 a	1.0±0.2a	0.9±0.2 a	16.0±1.1 a	4.3±1.1 a	1.0±0.4 a	2.2±0.5 a	0.6±0.1 a
Tame grassland	14.3±1.6 a	1.5±0 a	0.7±0 a	0.7±0 a	1.3±0.2 a	14.8±1.9 a	2.7±0.4 a	0.6±0.1 a	1.3±0.3 a	0.6±0.1 a
Annual Crop	13.3±0.7 a	1.4±0.2 a	0.6±0.2 a	0.8±0 a	1.2±0.3 a	11.9±0.6 a	3.3±0.8 a	0.8±0 a	2.7±0.7 a	0.6±0.1 a

[a] orthophosphate (OrthP), pyrophosphate (Pyro), polyphosphate (Poly), phosphonates (Phos), *myo*-inositol hexakisphosphate (myo-IHP), *scyllo*-inositol hexakisphosphate (Scy-IHP), *neo*-inositol hexakisphosphate (neo-IHP), *chiro*-inositol hexakisphosphate 4e/2a (Chiro-IHP1), *chiro*-inositol hexakisphosphate 2e/4a (Chiro-IHP2), a and b glycerophosphate (a-glyc and b-glyc, respectively), mononucleotides (Nucl), choline phosphate (Pchol), glucose 1-phosphate (G1P), glucose 6-phosphate (G6P), orthophosphate monoesters, regions 1, 2, and 3 (Mono1, Mono2, and Mono3, respectively), deoxyribonucleic acid (DNA), and orthophosphate diesters, regions 1 and 2 (OthDi1and OthDi2, respectively).

[b] Values in each column followed by the same lowercase letters are not significantly different according to LSD ($P < 0.05$)

Table S7. Effect of land use type on the composition of the soil microbial community and phosphorus (P) cycling related functional genes based on permanova (adonis function).

	Factor	Pr (>F)	r^2
Archaeal community	Land use type	0.31	
Bacterial community	Land use type	<0.0001	0.57
Fungal community	Land use type	<0.0001	0.54
P cycling bacterial community	Land use type	<0.0001	0.50
P cycling functional genes	Land use type	0.0017	0.57
